# Serinc2 Drives the Progression of Cervical Cancer Through Regulating Myc Pathway

**DOI:** 10.1002/cam4.70296

**Published:** 2024-10-17

**Authors:** Xiaoping Wang, Chen Jiang, Qing Li

**Affiliations:** ^1^ Department of Obstetrics and Gynecology Jinan Maternity and Child Care Hospital Jinan Shandong People's Republic of China

**Keywords:** cell proliferation, cervical cancer, glycolysis, Myc, Serinc2

## Abstract

**Background:**

As one of the most common malignancies, cervical cancer (CC) seriously affects women's health. This study aimed to investigate the biological function of Serinc2 in CC.

**Methods:**

Serinc2 expression was surveyed utilizing immunohistochemistry, western blot, and qRT‐PCR. CC cell viability, invasion, proliferation, migration, and apoptosis, were detected via CCK‐8, Transwell assay, colony formation, wound healing assay, and flow cytometry. Glucose consumption, lactate production, and ATP levels were determined by the corresponding kit. The protein expression of c‐Myc, PDK1, HK2, PFKP, LDHA, Snail, Vimentin, N‐cadherin, and E‐cadherin was detected via western blot. The interaction between the promoter of PFKP and Myc was confirmed through luciferase reporter assay and Chip assay. In vivo, to evaluate the function of Serinc2 on tumor growth, a xenograft mouse model was used.

**Results:**

In CC tissues and cells, Serinc2 was upregulated. In CC cells, knockdown of Serinc2 suppressed cell invasion, proliferation, migration, decreased the expression of Snail, Vimentin, N‐cadherin, HK2, PFKP, LDHA, and PDK1, increased E‐cadherin expression, reduced glucose consumption and the production of lactate and ATP, and induced cell apoptosis; Serinc2 overexpression led to the opposite results. Mechanically, Serinc2 promoted Myc expression, and Myc induced PFKP expression. Furthermore, overexpressed Myc abolished the inhibitive influences of Serinc2 knockdown on the malignant behaviors of CC cells. Additionally, knockdown of Serinc2 inhibited tumor growth and reduced the protein expression of c‐Myc, PFKP, LDHA, and PDK1 in vivo.

**Conclusions:**

Knockdown of Serinc2 inhibited the malignant progression of CC, which was achieved via Myc pathway. Our study provides novel insight into CC pathogenesis.

## 1 Introduction

As a common cancer, cervical cancer (CC) possesses a high incidence and mortality rate, which imposes a significant burden on the public health system [1, 2]. The etiology of CC is complex, and its occurrence is related to many factors, such as genetic changes, unhealthy living habits, human papillomavirus (HPV) infection, and biochemical alterations [3, 4, 5]. At present, for preventing early CC, vaccination and regular screening have been used [6]. However, most patients with CC are found in late stage, resulting in a poor prognosis [7, 8]. Currently, the main therapeutic methods of CC include surgery, targeted therapy, chemotherapy, and radiotherapy [9]. Moreover, radiotherapy and chemotherapy not only have an effect on cancer cells but also have a significant killing effect on normal cells, leading to easy metastasis and recurrence of CC [3, 10]. Therefore, studying the molecular mechanism of CC is urgent for development of new therapy methods.

As a member of Serinc family, serine incorporator 2 (Serinc2) participates in the incorporation of serine and membrane [11]. Serinc2 has a hand in the progression of various cancers. For example, in lung adenocarcinoma, cell invasion, proliferation, and migration were suppressed by Serinc2 knockdown [12]. High Serinc2 expression is related to the shorter overall survival (OS) in low‐grade glioma, indicating that Serinc2 may be a potential prognostic marker [13]. In papillary thyroid carcinoma (PTC), Serinc2 expression is enhanced and Serinc2 may be a tumor‐driven indicator [14]. Moreover, SEELA‐Serinc2 axis participates in metabolic regulation, thereby affecting the progression of leukemia [15]. However, the influences of Serinc2 remain unclear in CC.

Herein, we investigated the influences of Serinc2 on biological behavior of CC cells. In vivo, the effect of Serinc2 knockdown on tumor growth was investigated. In addition, the potential molecular mechanisms related to Serinc2 were explored.

## 2 Methods

### 2.1 Bioinformatics Analysis

In normal and CC tissue, Gene Expression Profiling Interactive Analysis 2 (GEPIA2) was exploited to analyze Serinc2 expression. The survival of patients with CC was assessed by Kaplan‐Meier plotter based on the Cancer Genome Atlas (TCGA) database. Gene set enrichment analysis (GSEA) was performed to seek the biological processes associated with Serinc2. The hTFtarget predicted the binding of PFKP promoter and Myc.

### 2.2 Specimen Collection

The ethics committee of our hospital approved this study. Twenty‐five pairs of CC and normal tissues were collected. Gynecologic pathologist identified all tissues. Before surgery, all patients didn't receive any treatment, such as biotherapy, chemotherapy, and radiotherapy. All patients provided informed written consent.

### 2.3 Cell Culture and Transfection

Human cervical epithelial cells (HcerEpic) were purchased from ATCC (USA), and the cells were cultured in cervical epithelial cell basal medium (ATCC). Procell (Wuhan, China) provided CC cells (C‐33A, CaSki, and SiHa). Minimum essential medium (Procell) supplemented with 1% penicillin/streptomycin (P/S, Procell) and 10% fetal bovine serum (FBS; Gibco, Carlsbad, CA, USA) was applied to culture C‐33A and SiHa cells. CaSki cells were cultured in RPMI‐1640 (BasalMedia, Shanghai, China) with 1% P/S and 10% FBS. At 37°C under 5% CO_2_, all cells were maintained.

Small interfering RNAs (siRNAs) targeting Serinc2 and Myc (siSerinc2‐1, siSerinc2‐2, siMyc‐1, and siMyc‐2), siRNA negative control (siNC), vector, and overexpressing vector of Serinc2 and Myc were provided by GenePharma (Shanghai, China). For transfection, Lipofectamine 2000 reagent (Invitrogen, USA) was used.

### 2.4 Quantitative Reverse Transcription‐Polymerase Chain Reaction (qRT‐PCR)

To extract total RNA, TRIzol reagent (Invitrogen) was applied. Next, cDNA was synthesized via the PrimeScript RT reagent kit (Takara, Japan). QRT‐PCR was carried out using the ChamQ SYBR qPCR Master Mix (Vazyme, China). Based on the 2^−ΔΔCt^ method, the relative mRNA level was calculated and GAPDH was selected as an internal control. The primer sequences were as follows: Serinc2, forward primer: 5′‐CAGCTCTACAAGCTGCCCTG‐3′ and reverse primer: 5′‐AATGTAGAAGGCACCCACGG‐3′; GAPDH, forward primer: 5′‐GAGAGAAACCCGGGAGGCTA‐3′, and reverse primer: 5′‐CCCAATACGACCAAATCCGTTG‐3′.

### 2.5 Western Blot

RIPA lysis buffer (Sigma‐Aldrich, St. Louis, MO, USA) was utilized to extract total protein. Protein samples (40 μg) were separated in SDS‐PAGE and then transferred to PVDF membrane (Millipore, Germany). At 4°C, membranes were incubated with primary antibodies (Serinc2, GAPDH, E‐cadherin, N‐cadherin, Vimentin, Snail, HK2, PFKP, PDK1, LDHA, and c‐Myc) overnight. Secondary antibody was applied to incubate with membranes for 2 h. Lastly, the blots were detected using ECL chemiluminescence detection kit (Vazyme). Image J software (National Institutes of Health, USA) was applied to analyze the relative protein level.

### 2.6 Detection of Cell Viability

After culturing for 0, 24, 48, and 72 h, transfected cells were cultured with 10 μL Cell Counting Kit‐8 (CCK‐8) solution (Beyotime Biotechnology, Shanghai, China) for 2 h. At 450 nm, the microplate reader was utilized to monitor optical density (OD).

### 2.7 Colony Formation Assay

Transfected cells were cultured in the 6‐well plates for 2 weeks. After fixing with 4% paraformaldehyde (Solarbio, Beijing, China), colonies were stained with 0.1% crystal violet (Beyotime Biotechnology). Under a microscope, colonies were observed.

### 2.8 Detection of Cell Apoptosis

After collecting, transfected cells were resuspended in Annexin V‐FITC binding buffer (Beyotime Biotechnology). Afterwards, cells were incubated with Annexin V‐FITC and PI in dark for quarter of an hour. Cell apoptosis was assessed using flow cytometry.

### 2.9 Wound Healing Assay

Transfected cells were cultured in the 6‐well plates until they reached 100% confluency. Subsequently, in the monolayer of the cells, a scratch was generated using a pipette tip. After washing with phosphate buffer saline (PBS, Solarbio), in serum‐free medium, cells were cultured for 48 h. At 0 and 48 h, images were photographed. At the end, wound healing rate was calculated.

### 2.10 Transwell Assay

Transfected cells suspended in serum‐free medium were added into the upper of transwell chamber (Corning, USA) coated with Matrigel (BD Biosciences, USA). Medium containing 10% FBS was added to the lower of transwell chamber. After culturing for 48 h, under a light microscope (Olympus, Tokyo, Japan), the images of invaded cells were photographed.

### 2.11 Assessment of the Production of Lactate and Adenosine Triphosphate (ATP) and Glucose Consumption

In line with the instructions of the manufacturer, glucose uptake colorimetric assay kit (MAK083, Sigma‐Aldrich), lactate assay kit (K607‐100, BioVision, Milpitas, CA, USA), and ATP colorimetric/fluorometric assay kit (K354‐100, BioVision) were applied to determine glucose consumption, lactate production, and ATP level, respectively.

### 2.12 Measurement of Oxygen Consumption Rate (OCR) and Extracellular Acidification Rate (ECAR)

The Seahorse XFe96 analyzer (Seahorse Bioscience, USA) was applied to measure the changes of ECAR and OCR. Transfected cells were seeded in XFe96 cell culture microplates (Agilent Technologies). For OCR measurement, at specific time points, oligomycin, FCCP, and rotenone/antimycin A were sequentially added to XF assay medium (Seahorse Bioscience). For ECAR measurement, at specific time points, glucose, oligomycin, and 2‐DG were sequentially added to the culture medium.

### 2.13 Chromatin Immunoprecipitation (Chip)

SiHa and C‐33A cells were treated with 1% formaldehyde (Sigma‐Aldrich) and treated using glycine (Beyotime Biotechnology). The chromatin was extracted and then sheared to 200–500 bp fragments using sonication. Next, the sheared chromatin was incubated with IgG antibody and anti‐Myc antibody overnight, and then incubated with protein G magnetic beads for 2 h. After washing the beads, the chromatin was eluted. At 65°C overnight, the cross‐linking was reversed. After extracting and purifying of DNA, qRT‐PCR was performed.

### 2.14 Luciferase Reporter Assay

Tsingke Biotechnology provided the reporter vector of wild‐type PFKP promoter (PFKP‐WT) and mutant PFKP promoter (PFKP‐MUT). Lipofectamine 2000 reagent was applied to transfect with vector, Myc, PFKP‐WT, or PFKP‐MUT to HEK‐293T cells. After transfection for 48 h, the dual‐luciferase reporter assay system (Promega, USA) was taken to determine the luciferase activity.

### 2.15 Xenograft Mouse Model

The protocol was approved by animal care and use committee of our hospital. GemPharmatech (Nanjing, China) provided female BALB/c nude mice. SiHa cells transfected with sh‐NC and sh‐Serinc2 were subcutaneously injected to the right flanks of mice. The experiment lasted for 28 days. During this period, tumor volume was measured every 4 days. Mice were sacrificed at 28‐days of post‐injection, and then the xenograft tumors were gained and weighed.

### 2.16 Immunohistochemistry (IHC) Assay

After fixing using 4% paraformaldehyde and embedding in paraffin, normal and tumor tissues were cut into sections. Next, the tissue sections were deparaffinized using xylene, followed by hydration with graded ethanol. After treating with H_2_O_2_ and blocking in bovine serum albumin (BSA, Beyotime Biotechnology), the tissue sections were handled with anti‐Serinc2 and anti‐Ki67 primary antibody and then handled with secondary antibody for 1 h. To stain and counterstain the sections, the 3′, 3′‐diaminobenzidine (DAB, Sigma) and hematoxylin were applied. Under a light microscope, the sections were observed.

### 2.17 Statistical Analysis

For statistical analysis and graphing, GraphPad Prism 9 software was used. For difference comparisons, the Student's *t*‐test and one‐way ANOVA were applied. The results were displayed as the mean ± standard deviation. When *p* < 0.05, the difference was considered as statistically significant.

## 3 Results

### 3.1 In CC Tissues and Cells, Serinc2 is Highly Expressed

**FIGURE 1:**
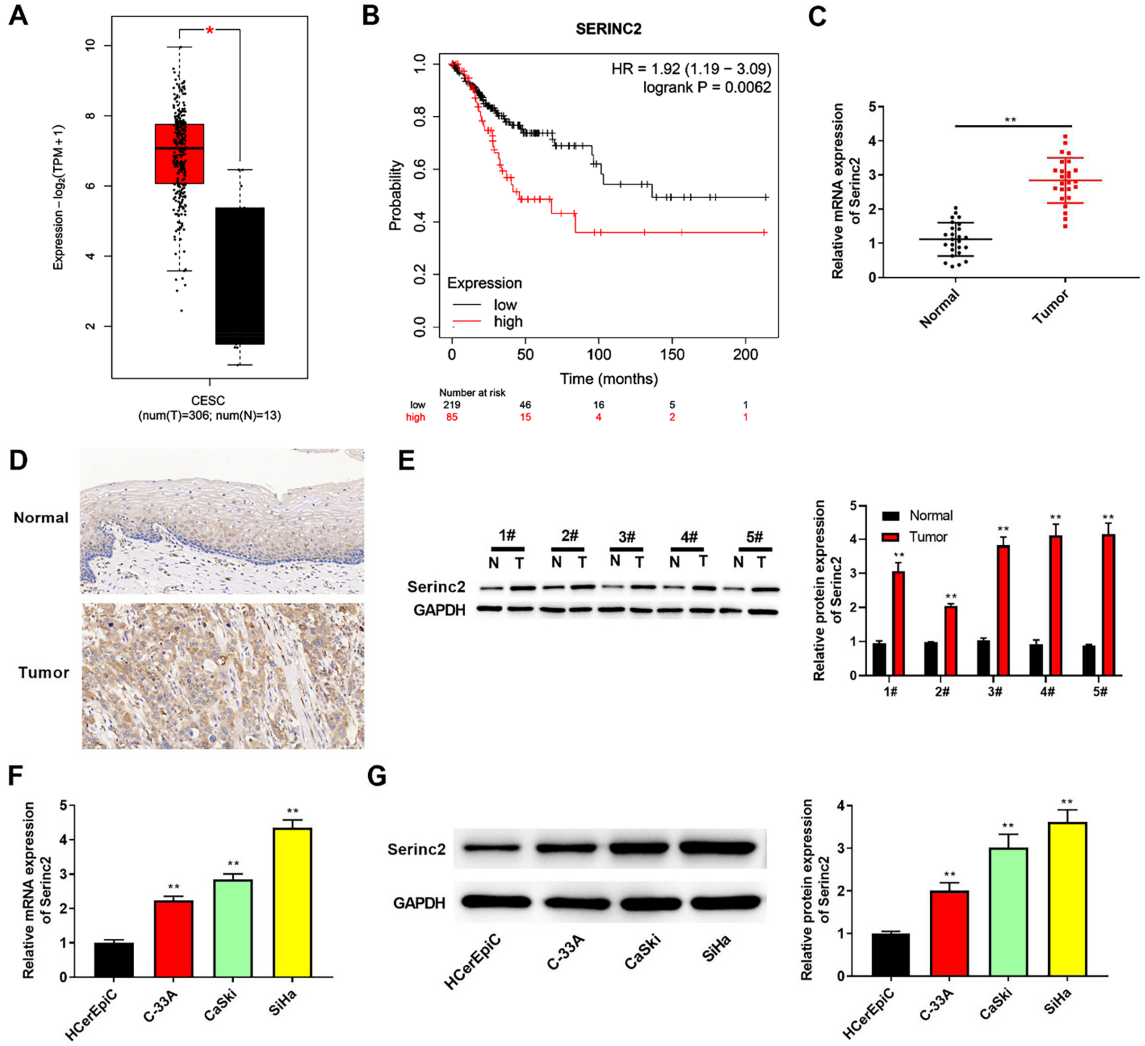
Serinc2 expression in CC tissues and cells. (A) Serinc2 expression was analyzed in normal and CC tissues using GEPIA2. (B) Based on TCGA database, in patients with CC, the prognosis of Serinc2 was analyzed via Kaplan–Meier plotter analysis. Serinc2 difference between normal and CC tissues was surveyed using qRT‐PCR(C), IHC (D), and western blot (E). Serinc2 expression was checked using qRT‐PCR (F) and western blot (G). Compared with normal tissues or HCerEpiC cells, **p < 0.01.

We analyzed Serinc2 expression in CC and normal tissues using GEPIA2. Figure 1A showed that Serinc2 expression was enhanced in CC tissues. Based on TCGA database, in patients with CC, Serinc2 high expression was found to have a bearing on the poor prognosis (Figure 1B). Afterwards, we collected the normal and CC tissues and detected Serinc2 expression. As presented in Figure 1C, in CC tissues, Serinc2 expression was elevated. This trend was further confirmed by IHC and western blot (Figure 1D,E). Interestingly, in CC cells (C‐33A, CaSki, and SiHa), Serinc2 expression was up‐regulated (Figure 1F,G).

### 3.2 Serinc2 Promotes CC Cell Proliferation and Suppresses Cell Apoptosis

**FIGURE 2:**
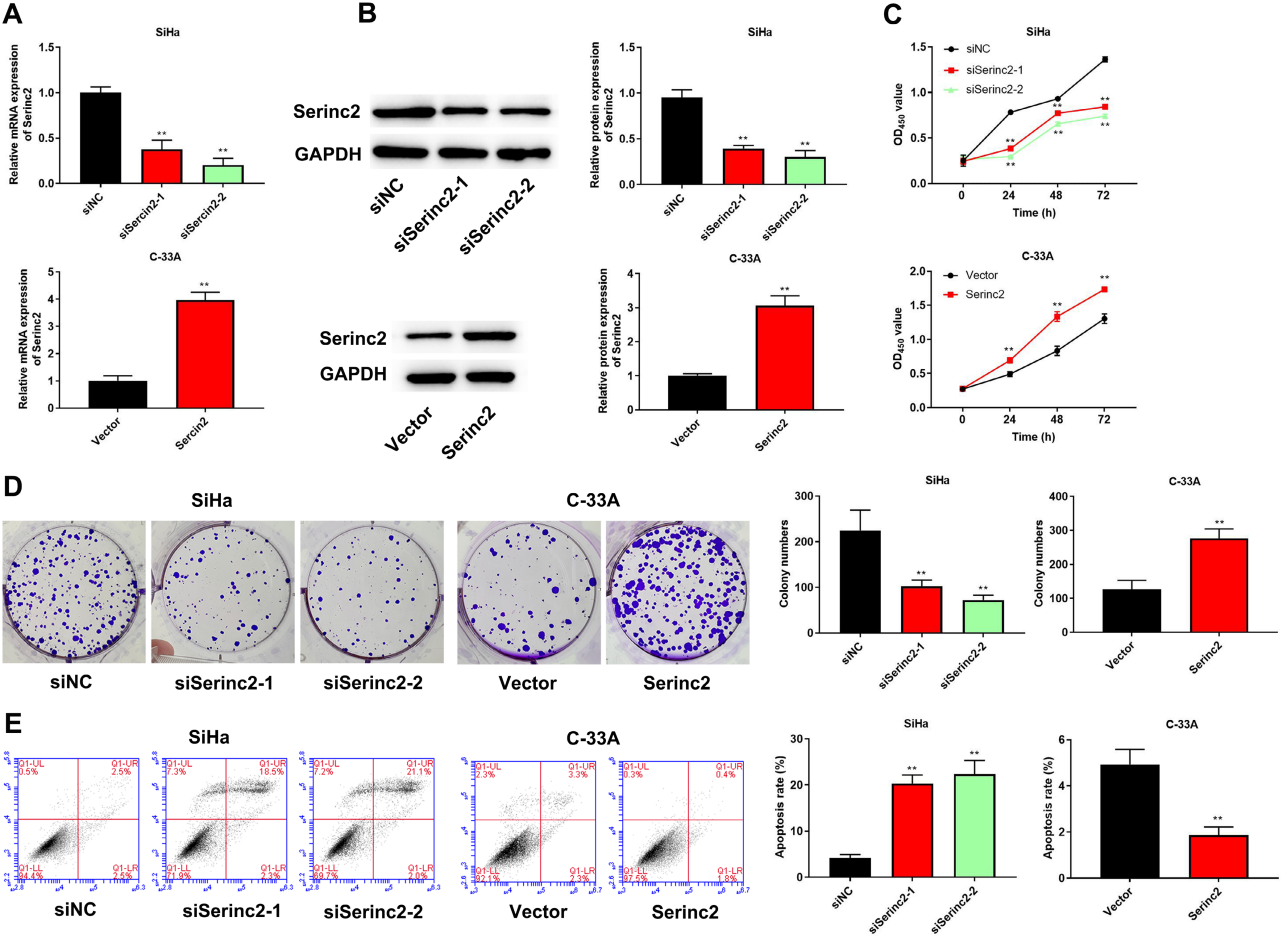
The effects of Serinc2 on CC cell proliferation and apoptosis. QRT‐PCR (A) and western blot (B) were applied to determine Serinc2 expression. (C) Cell viability was checked using CCK‐8 assay. (D) Cell proliferation was determined via colony formation assay. (E) Cell apoptosis was checked using flow cytometry. Compared with siNC or vector group, **p < 0.01.

To investigate Serinc2’ biological function in CC, Serinc2 was down‐expressed in SiHa cells with higher Serinc2 expression (transfected with siSerinc2‐1 and siSerinc2‐2) and overexpressed in C‐33A cells with lower Serinc2 expression (transfected with pcDNA3.1‐Serinc2). QRT‐PCR (Figure 2A) and western blot (Figure 2B) confirmed transfection efficiency. Figure 2C,D indicated that knockdown of Serinc2 decreased cell viability and inhibited colony‐forming, but Serinc2 overexpression enhanced cell viability and promoted colony‐forming. In addition, knockdown of Serinc2 increased SiHa cell apoptosis, while Serinc2 overexpression in C‐33A cells displayed the contrary effects (Figure 2E).

### 3.3 Serinc2 Promotes CC Cell Migration and Invasion

**FIGURE 3:**
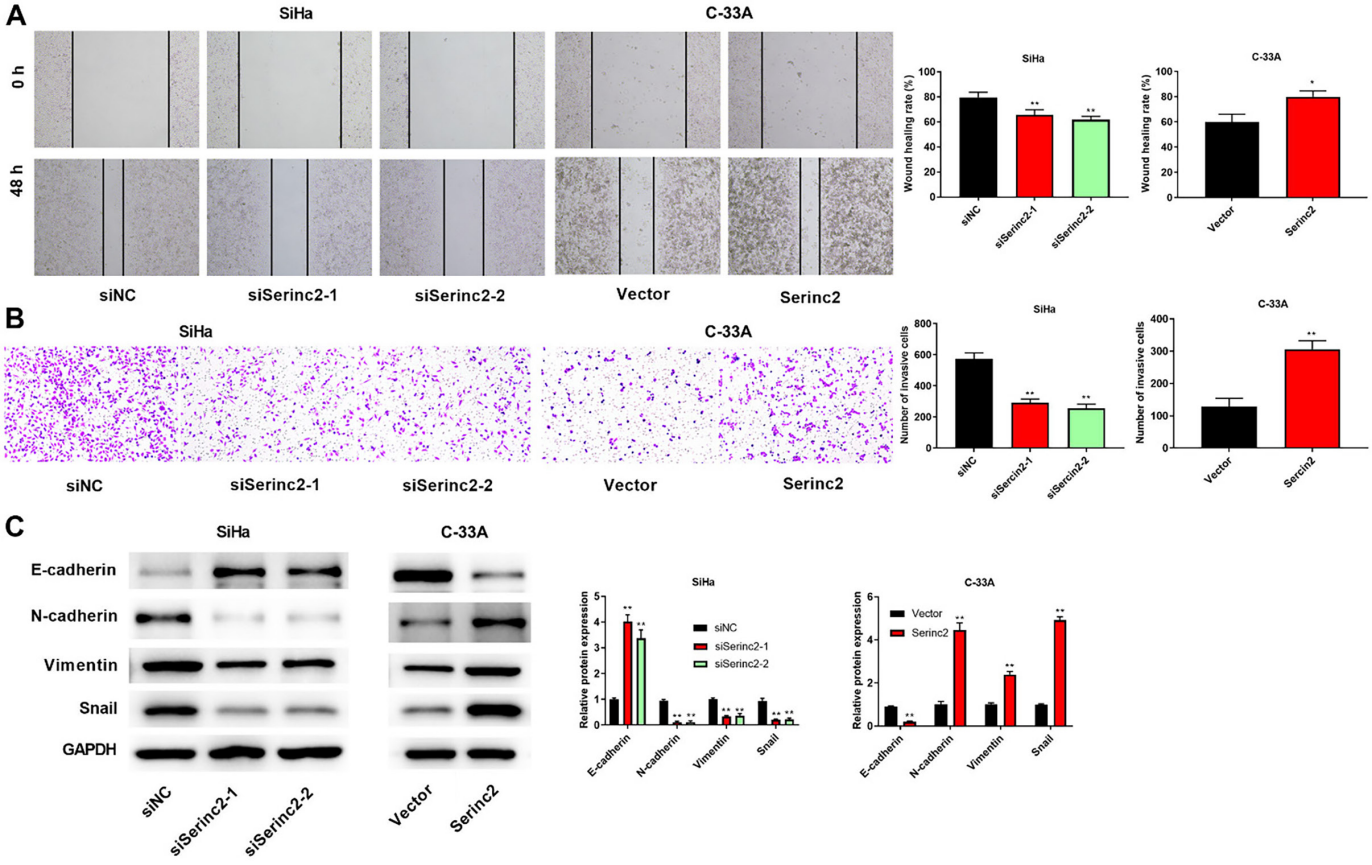
The effects of Serinc2 on CC cell migration and invasion. (A) Wound healing assay was utilized to check cell migration. (B) Cell invasion was checked via Transwell assay. (C) E‐cadherin, Snail, Vimentin, and N‐cadherin expression was checked using western blot. Compared with siNC or vector group, *p < 0.05 or **p < 0.01.

Figure 3A elucidated that knockdown of Serinc2 reduced wound healing rate, whereas Serinc2 overexpression resulted in the increased wound healing rate. Meantime, knockdown of Serinc2 decreased the number of invasive cell, but Serinc2 overexpression increased the number of invasive cell (Figure 3B). Moreover, knockdown of Serinc2 promoted E‐cadherin expression and inhibited Snail, Vimentin, and N‐cadherin expression; on the contrary, E‐cadherin expression was inhibited and Snail, Vimentin, and N‐cadherin expression was promoted by Serinc2 overexpression (Figure 3C).

### 3.4 Serinc2 Promotes Glycolysis

**FIGURE 4:**
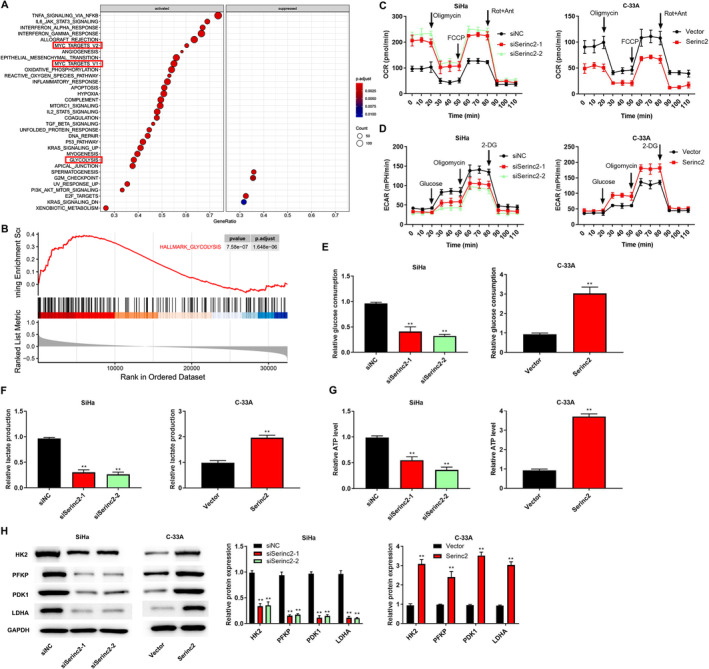
The effects of Serinc2 on glycolysis in CC cells. (A) GSEA analysis for Serinc2. (B) GSEA analysis indicated that Serinc2 activated the HALLMARK_GLYCOLYSIS. The levels of OCR (C), ECAR (D), glucose consumption (E), lactate production (F), and ATP (G) were measured. (H) HK2, PFKP, PDK1, and LDHA expression was surveyed using western blot. Comapred with siNC or vector group, **p < 0.01.

According to GSEA analysis, Serinc2 can regulate some biological processes, including glycolysis (Figure 4A,B). Figure 4C,D revealed that knockdown of Serinc2 increased OCR and decreased ECAR, but Serinc2 overexpression led to the contrary results. We assessed the changes of glycolysis‐related metabolites. Figure 4E–G showed that knockdown of Serinc2 reduced glucose consumption, lactate production, and ATP levels, while the increased trend of these metabolites was observed in cells with Serinc2 overexpression. In addition, the levels of PDK1, HK2, PFKP, and LDHA were reduced by knockdown of Serinc2, whereas Serinc2 overexpression enhanced the levels of these proteins (Figure 4H).

### 3.5 Serinc2 Promotes Myc Expression and Myc Induces PFKP Transcription

**FIGURE 5:**
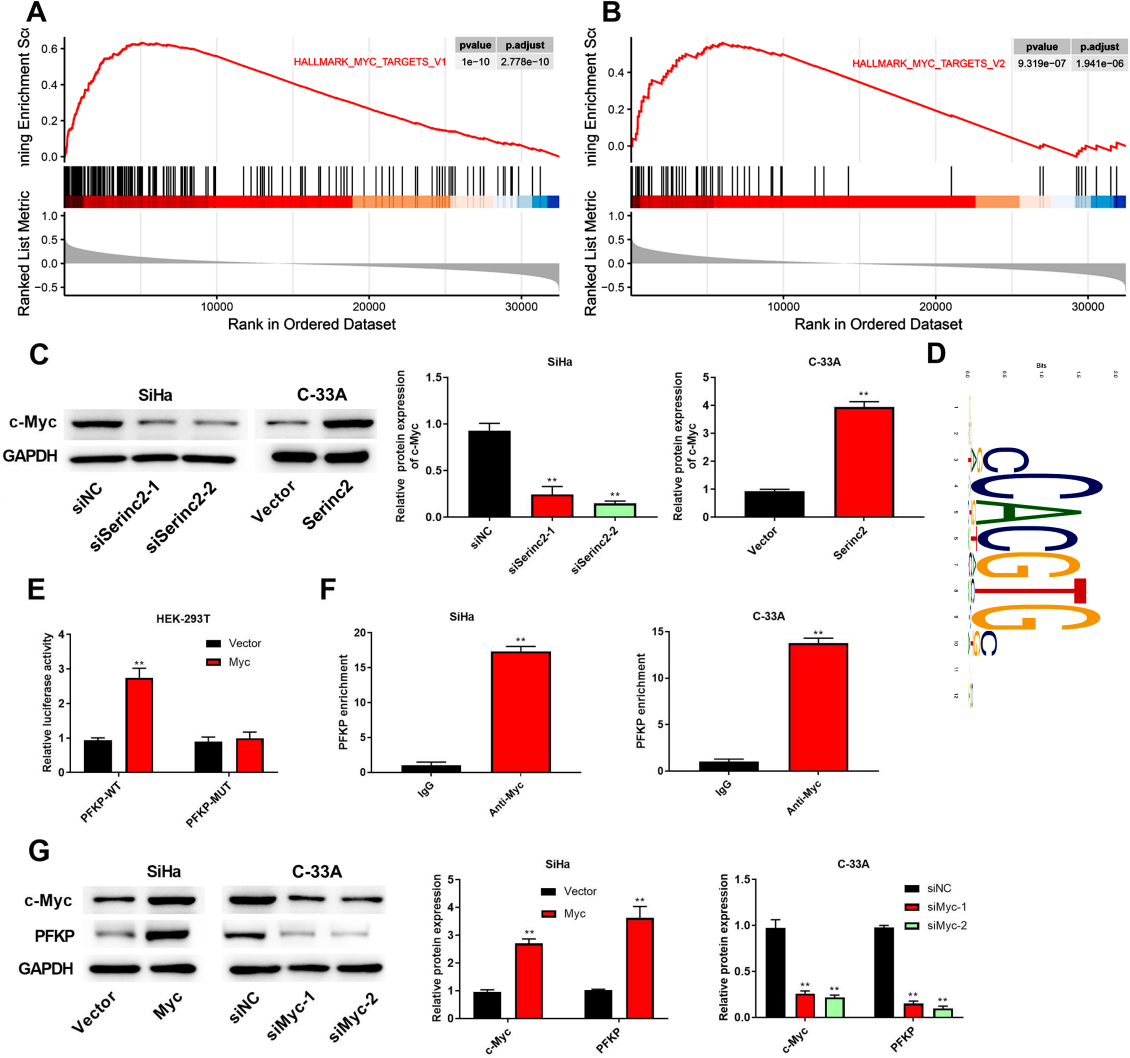
The effects of Serinc2 on Myc pathway. (A) GSEA analysis indicated that Serinc2 activated the HALLMARK_MYC_TARGETS_V1. (B) GSEA analysis indicated that Serinc2 activated the HALLMARK_MYC_TARGETS_V2. (C) c‐Myc expression was surveyed using western blot. (D) The motif of Myc. The relationship between Myc and PFKP promoter was identified through luciferase reporter assay (E) and Chip assay (F). (G) c‐Myc and PFKP expression was surveyed via western blot. Compared with siNC, vector, or IgG group, **p < 0.01.

GSEA analysis indicated that Serinc2 activated MYC targets (Figure 5A,B). Next, the changes of c‐Myc protein were investigated. As shown in Figure 5C, c‐Myc expression was reduced by knockdown of Serinc2, while Serinc2 overexpression increased c‐Myc expression. The hTFtarget predicted that PFKP might be regulated by Myc. Figure 5D presents the Myc motif. As displayed in Figure 5E, the luciferase activity of PFKP‐WT was enhanced by Myc. Anti‐Myc increased PFKP enrichment (Figure 5F), demonstrating that Myc could bind to PFKP promoter. Afterwards, SiHa cells were transfected with pcDNA3.1‐Myc and C‐33A cells were transfected with siMyc‐1 and siMyc‐2. As seen in Figure 5G, SiHa cells with pcDNA3.1‐Myc transfection showed a higher c‐Myc protein expression, and C‐33A cells transfected with siMyc‐1 and siMyc‐2 showed a lower c‐Myc protein expression. In addition, up‐regulated Myc expression promoted PFKP expression, but down‐regulated Myc expression led to a contrary tendency (Figure 5G).

### 3.6 Knockdown of Serinc2 Inhibits CC Cell Proliferation, Invasion, and Glycolysis Through Regulating Myc

**FIGURE 6:**
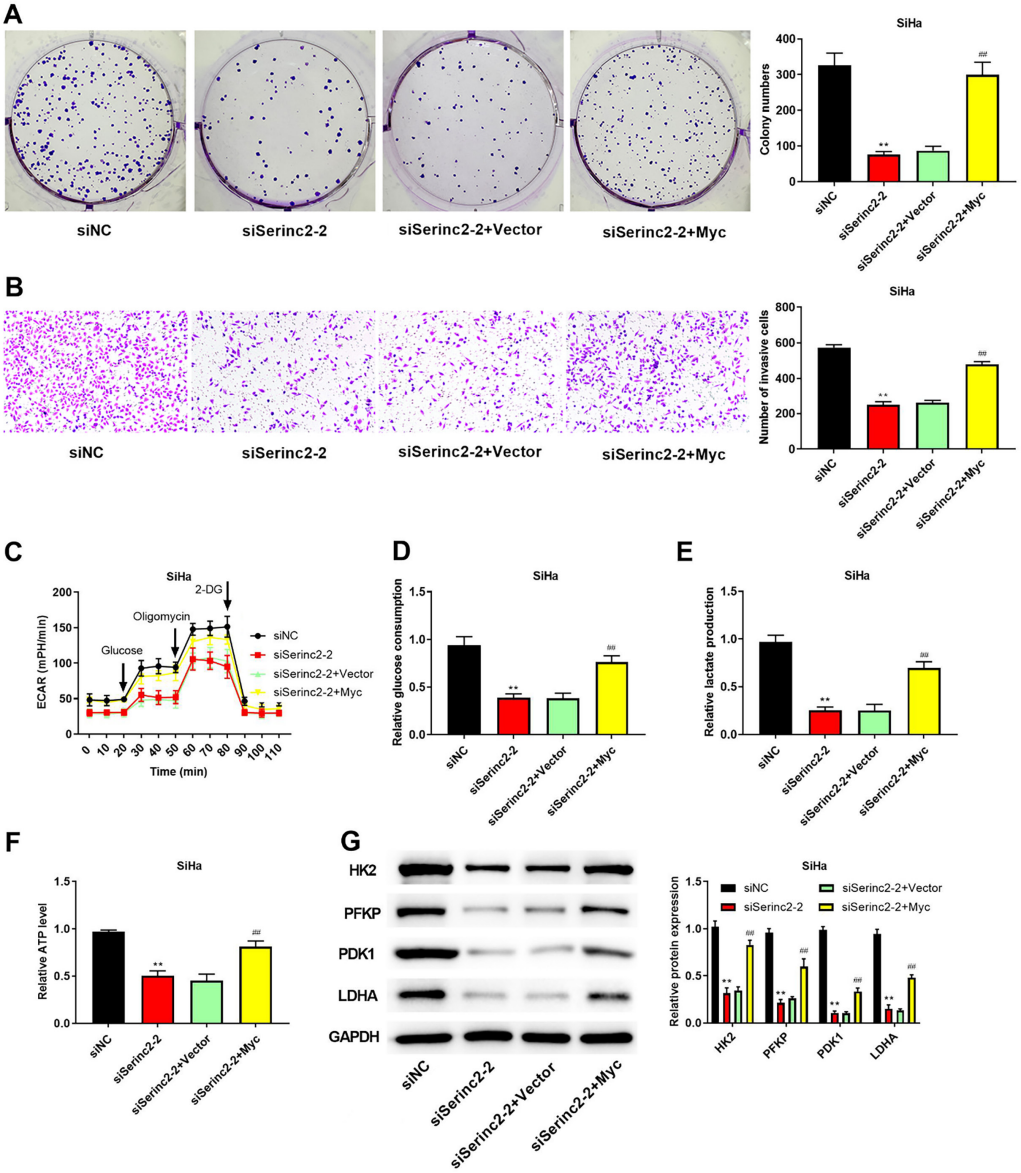
The role of Myc in knockdown of Serinc2 mediated tumor inhibiting effects. (A) Colony formation assay was applied to determine cell proliferation. (B) Transwell assay was utilized to check cell invasion. The levels of ECAR (C), glucose consumption (D), lactate production (E), and ATP (F) were measured. (G) HK2, PFKP, LDHA, and PDK1 expression was surveyed using western blot. Compared with siNC group, **p < 0.01; compared with siSerinc2‐2 + vector group, ^##^ *p* < 0.01.

To evaluate the role of Myc in Serinc2‐mediated regulation in CC, we elevated Myc expression and looked into cell proliferation, cell invasion, and glycolysis upon knockdown of Serinc2. Figure 6A,B showed that overexpressed Myc reversed the inhibition of Serinc2 knockdown on cell proliferation and invasion. Additionally, knockdown of Serinc2 reduced ECAR, glucose consumption, lactate production, and ATP, but Myc overexpression reversed this tendency (Figure 6C–F). Meanwhile, compared with siSerinc2‐2 + Vector group, overexpression of Myc increased the protein levels of HK2, PFKP, LDHA, and PDK1 (Figure 6G).

### 3.7 In Vivo, Knockdown of Serinc2 Restrains CC Tumor Growth

**FIGURE 7:**
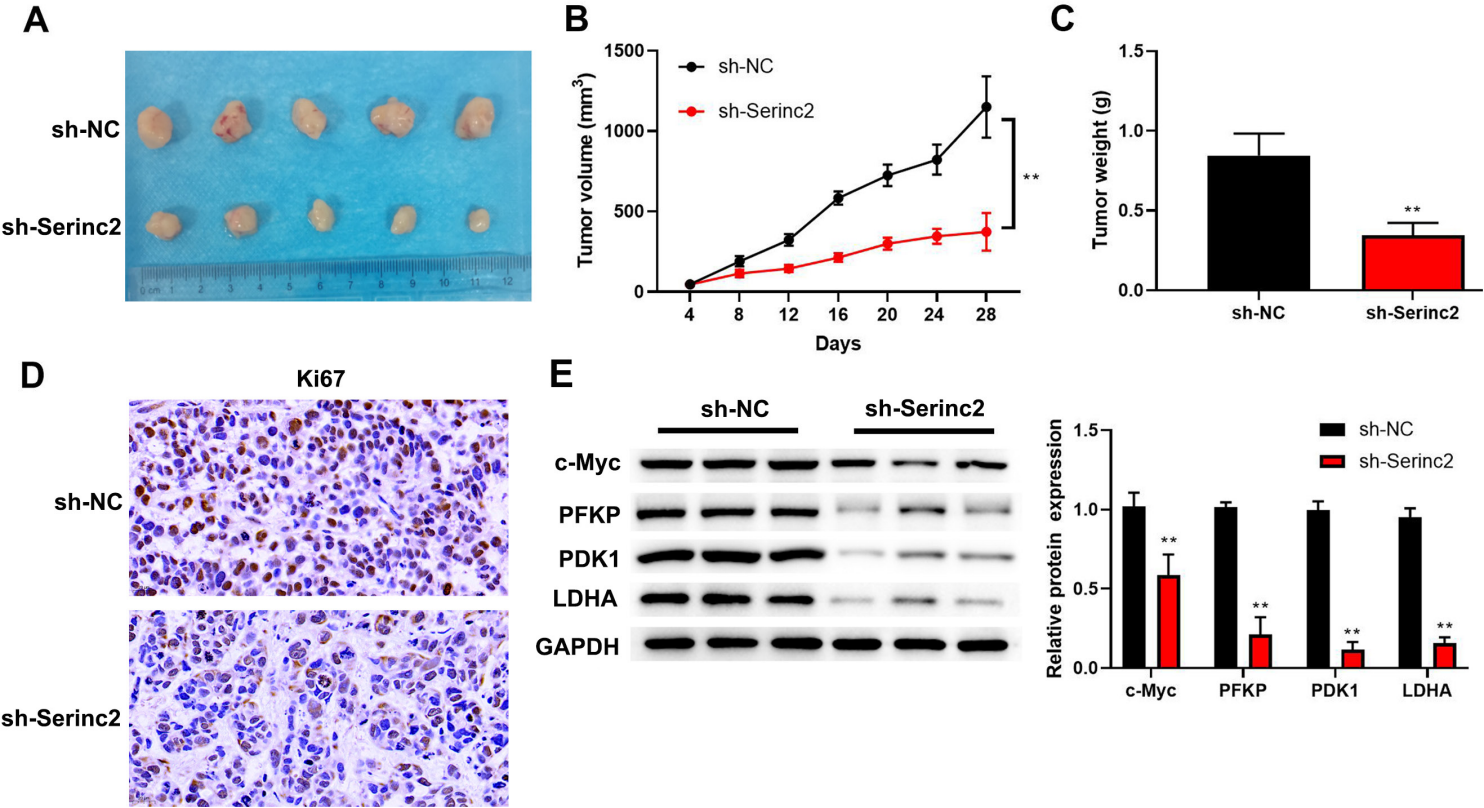
The effects of Serinc2 on CC tumor growth in vivo. (A) The images of xenograft tumors. The xenograft tumor volume (B) and weight (C) were calculated. (D) Ki67 expression was confirmed by IHC. (E) c‐Myc, PFKP, LDHA, and PDK1 expression was surveyed using western blot. Compared with sh‐NC group, **p < 0.01.

To further investigate the role of Serinc2 in CC progression, we constructed xenograft mouse model with low‐expression of Serinc2. A lower tumor volume and weight were observed in sh‐Serinc2 group (Figure 7A–C). IHC assay confirmed that low‐expression of Serinc2 reduced Ki67 expression (Figure 7D). Furthermore, c‐Myc, PFKP, LDHA, and PDK1 expression was decreased by low‐expression of Serinc2 (Figure 7E).

## 4 Discussion

In women, CC is a deadly malignant tumor [16]. Currently, for physicians and patients, the metastasis and recurrence of CC remain a big challenge [17]. Therefore, finding new targets and potential drugs is important for CC treatment. Tumor differentially expressed 2 (TDE2), also known as Serinc2, was first identified in non‐small cell lung cancer (NSCLC) cells, and TDE2 expression was higher in NSCLC samples [18]. Herein, based on TCGA database, in CC tissues, Serinc2 expression was enhanced. In patients with CC, high Serinc2 expression was related to the poor prognosis. Moreover, high Serinc2 expression was found in CC tissues and cells. Collectively, these findings suggested that Serinc2 might implicate in CC progression.

In numerous cancers, Serinc2 was confirmed to exert oncogenic function. Knockdown of Serinc2 inhibited cell invasion, migration, and proliferation via regulating the PI3K/AKT pathway in lung adenocarcinoma [12]. Low‐expression of Serinc2 inhibited cell proliferation and induced cell apoptosis in PTC [14]. In this study, through functional assays, overexpression of Serinc2 promoted CC cell invasion, proliferation, and migration, and inhibited cell apoptosis, while the contrary results were observed in cells with Serinc2 knockdown. Moreover, in vivo, low‐expression of Serinc2 restrained CC tumor growth. In malignant tumors, EMT is a common phenomenon. EMT is involved in various biological processes of CC, such as cancer stemness, drug resistance, immune escape, and metastasis [19, 20, 21, 22]. During EMT process, epithelial cells lose epithelial components and cell adhesion to acquire migratory and invasive phenotype [23, 24]. Mesenchymal markers include Vimentin and N‐cadherin; E‐cadherin is an epithelial marker [25]. As a EMT transcription factor, Snail plays a notable role in EMT [26]. Tang et al. revealed that shikonin inhibited EMT through upregulating miR‐183‐5p and inhibiting Snail, thereby restraining CC progression [27]. In our current study, knockdown of Serinc2 promoted E‐cadherin expression and inhibited Snail, Vimentin, and N‐cadherin expression, but the opposite results were observed in cells with Serinc2 overexpression. Taken together, these data indicated that low‐expression of Serinc2 inhibited cell migration, invasion, EMT, and tumor growth in CC.

In recent years, metabolic reprogramming has been considered as a hallmark of cancer cells [28]. In cancer cells, the most characteristic metabolic change is glycolysis (also known as Warburg effect), which increases glucose uptake and lactate production [29]. For cancer cell survival and proliferation, glycolysis supports energy requirements [30]. Currently, numerous studies have demonstrated that enhanced glycolysis promoted CC progression [31, 32, 33, 34]. Interestingly, in the present study, GSEA analysis found Serinc2 promoted glycolysis. Moreover, knockdown of Serinc2 reduced glucose consumption and the production of lactate and ATP, while the contrary results were observed in cells with Serinc2 overexpression. OCR is an indicator of mitochondrial oxidative respiration, and ECAR is an indicator of aerobic glycolysis [35]. Here, we found that knockdown of Serinc2 increased OCR and decreased ECAR, but Serinc2 overexpression led to the contrary results. What is more, the lower expression of key glycolysis proteins (PDK1, HK2, PFKP, and LDHA) was observed in cells with Serinc2 knockdown, whereas Serinc2 overexpression enhanced the expression of these proteins. Moreover, in vivo, low‐expression of Serinc2 inhibited PFKP, LDHA, and PDK1 protein expression. All in all, Serinc2 promoted glycolysis in CC.

In our study, GSEA analysis showed that Serinc2 activated MYC targets. Meantime, Serinc2 overexpression increased c‐Myc expression, but c‐Myc expression was reduced by knockdown of Serinc2. In vivo experiments further validated that low‐expression of Serinc2 inhibited c‐Myc expression. As a key member of Myc oncogene family, c‐Myc has a hand in differentiation, proliferation, apoptosis, metabolism, and metastasis in cancer [36]. In CC, recent reports have shown that the increased c‐Myc expression is often observed [37, 38]. In cancer cells, c‐Myc overexpression promotes glycolysis and increases the energy source, thereby promoting cell invasion and proliferation [39, 40, 41]. In glycolysis, PFKP catalyzes the conversion of fructose‐6‐phosphate to fructose‐1,6‐bisphosphate [42]. In patients with CC, a previous study has shown that high PFKP expression has a hand in poor prognosis [43]. Herein, we found PFKP directly interacted with Myc. Moreover, up‐regulated Myc expression promoted PFKP expression, but down‐regulated Myc expression reduced PFKP expression. The following mechanism study showed that overexpressed Myc abolished the influences of Serinc2 knockdown on ECAR, glucose consumption, lactate production, ATP, glycolysis‐related proteins, cell proliferation, and cell invasion. In a word, these evidences uncovered that CC cell proliferation, invasion, and glycolysis were restrained by Serinc2 knockdown, which has a bearing on Myc.

## 5 Conclusion

In CC tissues and cells, Serinc2 was overexpressed. Overexpression of Serinc2 promoted CC cell invasion and glycolysis. Moreover, Serinc2 knockdown inhibited CC cell proliferation and tumor growth. The mechanism underlying the role of Serinc2 in CC progression might be related to Myc pathway. For CC treatment, Serinc2 may be a promising therapeutic target.

## Author Contributions

**Xiaoping Wang:** conceptualization (equal), data curation (equal), formal analysis (equal). **Chen Jiang:** formal analysis (equal). **Qing Li:** data curation (equal), formal analysis (equal).

## Ethics Statement

The experimental protocol of our study was performed in accordance with the Guide for the Care and Use of Laboratory Animals and approved by Jinan Maternity and Child Care Hospital. The study followed ARRIVE guidelines.

## Consent

The protocol of this research has been approved by the Ethics Committee of Jinan Maternity and Child Care Hospital. The research complied with the Declaration of Helsinki. All patients have signed written informed consent.

## Conflicts of Interest

The authors declare no conflicts of interest.

## Data Availability

The datasets used and analyzed during the current study are available from the corresponding author on reasonable request.
